# Molecular Modeling and Dynamics of a Complete Connexin-43 Gap Junction Channel in Various Phosphorylation States

**DOI:** 10.1021/acs.jpcb.6c00338

**Published:** 2026-03-16

**Authors:** Ya Gao, Jian Zuo, Matthias M. Falk, Wonpil Im

**Affiliations:** 1 School of Mathematics, Physics and Statistics, 66323 Shanghai University of Engineering Science, Shanghai 201620, China; 2 Department of Biological Sciences, 1687 Lehigh University, Bethlehem, Pennsylvania 18015, United States

## Abstract

Gap junction channels, formed by the docking of two hemichannels from adjacent cells, are essential for intercellular communication. Connexin-43 (Cx43), the most widely expressed connexin, is critically involved in numerous physiological processes. Phosphorylation of Cx43 is a key regulatory mechanism that influences all aspects of its function, including trafficking, channel gating, and permeability. Here, we report a full-length computational model of the dodecameric Cx43 gap junction channel in double bilayers, including its intracellular loops and cytoplasmic regulatory C-terminal domains (CTDs). Furthermore, we performed all-atom molecular dynamics simulations of four systems representing different phosphorylation states. Our results demonstrate that increased phosphorylation of serine residues in the CTD induces more extended and flexible CTD conformations with greater solvent exposure, meanwhile narrowing the channel pore. Distinct gating states are closely associated with hydrophobic interactions between the N-terminal helices (NTHs) and transmembrane domain 2 (TM2). Unfolding of the NTHs disrupts the interactions, leading to pore distortion and a transition from the initial closed state to a more open conformation. These findings provide novel insights into the structural dynamics and regulatory mechanisms of the Cx43 gap junction channels.

## Graphical abstract

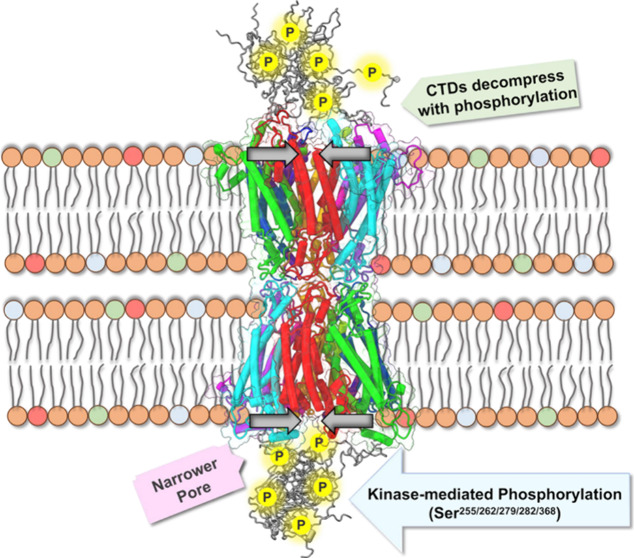

## Introduction

Gap junction channels (GJCs), formed by the docking of two opposing hemichannels (connexons) from adjacent cells,^1^ facilitate direct intercellular communication by creating a pore that provides a pathway for electrical and metabolic signaling between cells.^2–7^ Thus, GJCs play crucial roles in diverse biological processes, including cardiac contraction, electron coupling, cell differentiation during development, and programmed cell death.^6,8^ Each hemichannel is composed of six connexins (Cxs), which are transmembrane proteins that are widely expressed in many animal tissues. Each connexin protein has four α-helical transmembrane domains (TM1 to TM4), two antiparallel oriented extracellular loops (ECL1 and ECL2) connecting TM1–TM2 and TM3–TM4, respectively, an N-terminal helix (NTH) domain that folds into the channel vestibule and is connected to the pore-lining TM1 via a short linker, an intracellular loop (ICL) connecting TM2-TM3, and the cytoplasmic C-terminal domain (CTD).^9–11^ In humans, there are 21 distinct connexin isoforms^12^ that are oligomerized and transported to the plasma membrane. Among these, 43 kDa connexin-43 (Cx43, gene name *GJA1*) is the most widely expressed connexin. It is found in the majority of cell types and is the best studied connexin protein.^13^ Thus, cell coupling via Cx43 GJCs is important in a wide range of cellular processes. Mutations or dysregulation of Cx43 is associated with a variety of human diseases, including oculodentodigital dysplasia, palmoplantar keratoderma, heart disease, and various cancers.^14^

GJC function is controlled by various mechanisms among which phosphorylation is the most common means, and a large body of literature describes its role in regulating all aspects of GJC-mediated intercellular communication (GJIC) and its structure and function in health and disease.^15–19^ In particular, Cx43 undergoes phosphorylation at multiple sites predominantly in its CTD, with at least 21 identified phosphorylation residues^20^ comprising 19 serine and 2 tyrosine residues, and its phosphorylation state has been found to be regulated by the action of more than 10 kinases and phosphatases. For example, protein kinase C (PKC) has been demonstrated to phosphorylate Cx43 at Ser368 in vitro, while treatment with tissue polypeptide antigen (TPA) leads to decreased gap junctional communication and promotes phosphorylation at Ser262 and Ser368.^21–24^ Additionally, mitogen-activated protein kinase (MAPK) via different growth factors also phosphorylates Cx43 at Ser255/279/282,^25–27^ resulting in the downregulation of GJC communication. Recent studies in the author’s and other laboratories have shown that phosphorylation on these five serine residues plays a major role in GJC internalization and degradation.^28–35^ Together, extensive studies indicate that Cx43 is a highly phosphorylated and tightly controlled protein, with its phosphorylation serving as a fundamental mechanism governing all aspects of its trafficking, assembly, and function.

Despite the critical role of Cx43 GJCs in human health and disease, the molecular basis underlying the function and regulation of Cx43 GJCs remains incomplete, largely due to the lack of a complete high-resolution structure. The first, conceptual structure of GJCs was developed in 1977 based on high-resolution freeze fracture electron microscopy (EM) images.^36^ The first structural analysis of a recombinant cardiac α_1_[Cx43] GJC with an in-plane resolution of ∼7.5 Å was determined by cryo-EM in 1999,^37^ revealing that each connexon was formed by 24 closely packed TM α-helices, and how the ECLs form the docking interface between two hemichannels. More recently, Lee et al. performed cryo-EM single particle analyses of reconstituted Cx43 GJCs under various conditions, identifying three distinct NTH conformations coexisting in purified channel populations.^38^ Concurrently, Qi et al. reported an atomic resolution (2.26 Å) cryo-EM structure of the human Cx43 GJC,^39^ suggesting that the captured states of Cx43 are consistent with a putative closed state. Structures of other GJCs have also been determined. In 2009, the first and still only GJC, a human Cx26 GJC was crystallized,^40^ revealing for the first time the intricate architecture of a double-membrane spanning GJC at atomic resolution. Related work^41^ revealed a prominent density in the pore of each hemichannel, suggesting that the channel is blocked by a physical obstruction in a closed state and its activity may be regulated with a plug in the vestibule. Recent in situ characterization has provided significant new support for this concept by demonstrating that UNC-1/stomatin blocks the entrance of a related innexin-based GJ channel.^42^ The Cx26 GJC structure further provided a detailed view of the interactions between the two extracellular regions of adjoining connexons and suggested that the N-termini, lining the pore entrance and forming a funnel with a restricted diameter of 14 Å at the entrance of the pore, play an important role in channel gating.^40^ More recently, a large number of cryo-EM studies provided additional high-resolution structures of GJs, as well as related pannexin and innexin (the invertebrate gap junction proteins) channels. Cryo-EM studies of Cx46/Cx50 GJCs demonstrated that compared to the Cx26 crystal structure,^40^ it adopted a more stable open-state conformation stabilized by the NTH domain.^43,44^ In 2020, Lee et al. resolved the cryo-EM structure of the Cx31.3 hemichannel, which features a narrow pore (∼8 Å diameter) formed by six NTHs arranged horizontally to occlude the cytoplasmic gate in a semipermeable gate state.^45^ In 2023 and 2024, the structures of wild type and X-linked Charcot–Marie–Tooth (CMTX1)-causing Cx32,^46^ and the electrical synapse-forming Cx36 GJ channels were solved.^47,48^ Cryo-EM-based structures of all three related pannexin proteins 1–3 ^49–55^ and of innexins^56,57^ have recently been solved as well.

Despite this significant progress, no atomic resolution structure of any connexin, pannexin, or innexin available today includes the ICLs, nor the important intrinsically unstructured regulatory CTDs due to the limitations of the applied experimental methods and thus evaded structural resolution. Thus, the contribution of these domains critical to all aspects of GJC functions remains unresolved. Here, we report for the first time structural dynamics and functional properties of a complete computational representation of a Cx43 GJC, including ICLs and CTDs. In addition, in an attempt to gain evidence for our hypothesis that phosphorylation regulates access of enzymes and components required for GJC internalization, Cx43 GJCs with unphosphorylated CTD (P0) and phosphorylated at different serine residues (Serine 368 (P1); Serine 279/282/368 (P3); Serine 255/262/279/282/368 (P5)), all phosphorylations that previously have been shown to play critical roles in GJC internalization and degradation^33,58,59^ were modeled and simulated. We describe the effect of these phosphorylations on the structural dynamics of the Cx43-CTD, as well as the Cx43 GJC gating and permeability.

## Materials and Methods

### Construction of a Complete Cx43 Gap Junction Channel

Figure 1 shows the steps of constructing a full-length Cx43 GJC configuration. The CTD structure (residues 236 to 382) was obtained from the protein data bank (PDB ID: 1R5S)^60^ and fused to a Cx43 GJC core structure (PDB ID: 7Z22)^39^ after energy minimization. CHARMM-GUI Membrane Builder^61–65^ was used to generate a hexagonal mixed bilayer membrane with an outer leaflet (extracellular side) of POPC:PSM:CHOL = 2:2:1 (51, 50, and 25 for POPC, PSM, and CHOL, respectively) and an inner leaflet (intracellular side) of POPC:PSM:POPS:CHOL = 4:3:10:3 (12, 9, 30, and 9 for POPC, PSM, POPS, and CHOL, respectively) for the full-length Cx43 hemichannel model. The lipid types and their respective ratios were chosen to ensure that the structural and dynamic properties of the simulated bilayer closely mimic those of mammalian plasma membranes.^66,67^ Under the applied hexagonal periodic boundary conditions, the distance between the GJC centers in the primary and neighboring image cells was ∼110 Å, similar to those of native GJCs in negatively stained EM images.^68^ Four initial GJC models with different phosphorylation sites, Cx43-0P (no Ser phosphorylation), Cx43-1P (Ser368 phosphorylated), Cx43-3P (Ser279/282/368 phosphorylated), and Cx43-5P (Ser255/262/279/282/368 phosphorylated), were computationally constructed. The missing ICL was modeled using CHARMM-GUI based on internal coordinates of the Cx43 GJC structure. A hexagonal TIP3P water box^69,70^ was added to mimic the solvent environment and meanwhile keep the number of water molecules at a minimum. K^+^ and Cl^–^ ions were added to neutralize the charge of the entire system and maintain a salt concentration of ∼0.15 M. Each system was then duplicated and rotated along the membrane normal axis (the *Z*-axis) to form a conformational GJC representation. For the complete modeled Cx43 GJC structure, we obtained a hydrophobic thickness of ∼27 Å for each membrane of the hemichannel, which is defined by the distance of C2 or C3 atoms (glycerol backbone carbon atoms) in each lipid between the inner and outer leaflets. The averaged connexon length, including C-terminal region was ∼150 Å, and the averaged intercellular gap distance was ∼14 Å.

**Figure 1:**
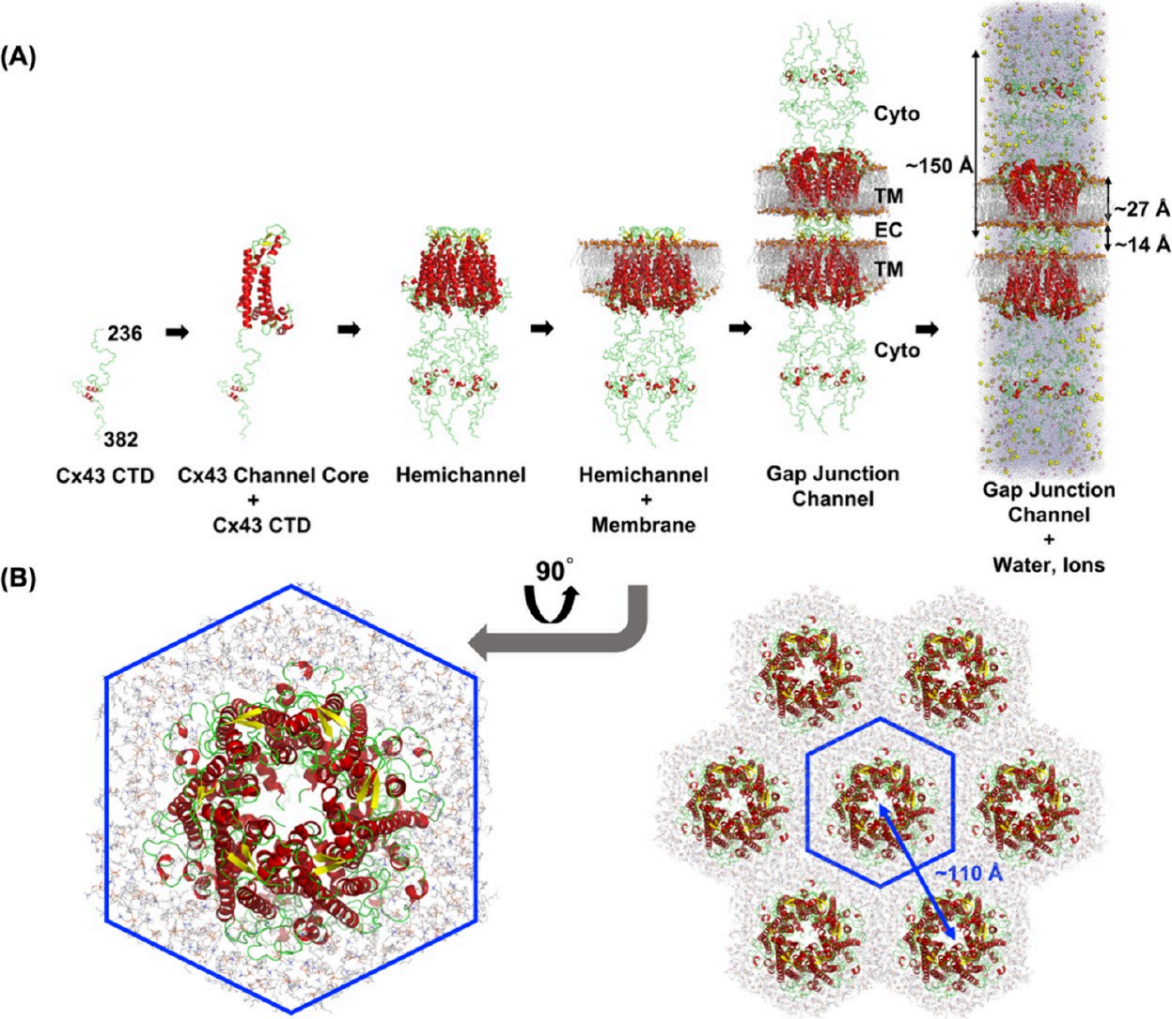
Building a full-length Cx43 gap junction channel configuration including its intracellular loop and unstructured C-terminal domains. (A) The Cx43 transmembrane and other α-helixes are colored in red, β-sheets are colored in yellow, and loops are colored in green. Lipid bilayers are represented with gray sticks, and to help with orientation, the sulfur atoms in lipids are highlighted with orange spheres. K^+^ and Cl^–^ ions are shown as yellow and pink spheres, respectively. Water molecules are represented with light-blue dots. The cytosolic (Cyto) domains (ICLs and CTDs), transmembrane (TM) domains, and ECLs are labeled. The averaged membrane hydrophobic thickness, connexon length, and intercellular gap length are labeled in the complete structural representation on the right. (B) Hexagonal arrangement of primary and neighboring images for Cx43-0P GJCs. The central blue hexagon outlines the primary image, while the surrounding structures represent the neighboring images. The arrow indicates the center-to-center spacing between adjacent images (∼110 Å).

### Modeling and Simulations

During simulations, the CHARMM36­(m) force field^71–77^ was used for the protein and lipids. The van der Waals interactions were smoothly switched off over 10–12 Å by a force-based switching function,^78^ and the long-range electrostatic interactions were calculated using the particle-mesh Ewald method^79^ with a mesh size of ∼1 Å. All simulations were performed using the input files generated by CHARMM-GUI,^80,81^ and we used GROMACS2020.4 ^82^ for both equilibration and production with the LINCS algorithm.^83^ The temperature was maintained using a Nosé–Hoover temperature coupling method^84,85^ with a τ_t_ of 1 ps. For pressure coupling (1 bar), a semi-isotropic Parrinello–Rahman method^86,87^ with a τ_p_ of 5 ps and a compressibility of 4.5 × 10^–5^ bar^–1^ was used. During the equilibration run, NVT (constant particle number, volume, and temperature) dynamics was first applied with a 1 fs time step for 250 ps. Subsequently, the NPT (constant particle number, pressure, and temperature) ensemble was applied with a 1 fs time step (for 125 ps). During the equilibration, positional and dihedral restraint potentials were applied, and their force constants were gradually reduced. The production run was performed with a 2 fs time step without any restraint potential. To enhance sampling and verify simulation convergence, three replicas were conducted for each system, and each production run was extended to 1500 ns, ensuring sufficient conformational sampling.

## Results and Discussion

### Complete Cx43 Gap Junction Channel

The constructed Cx43 GJC conformation is shown in Figure 2, in which the CTD was linked with the channel core portion to form a complete Cx43 GJC representation, including all its protein domains. The missing ICL was modeled using CHARMM-GUI, as shown in Figure 2, indicating a partially folded α-helix structure from Arg101 to Gly150. The β-sheets in the extracellular domain of two hemichannels are closely interplayed at the intercellular gap interface. To confirm the structural robustness of our computational model, the channel core of the complete GJC was aligned with the cryo-EM structure published by Qi et al.^39^ (shaded in gray). The substantial overlapping of helix bundles between the modeled and the cryo-EM structures confirms the reliability of our model.

**Figure 2:**
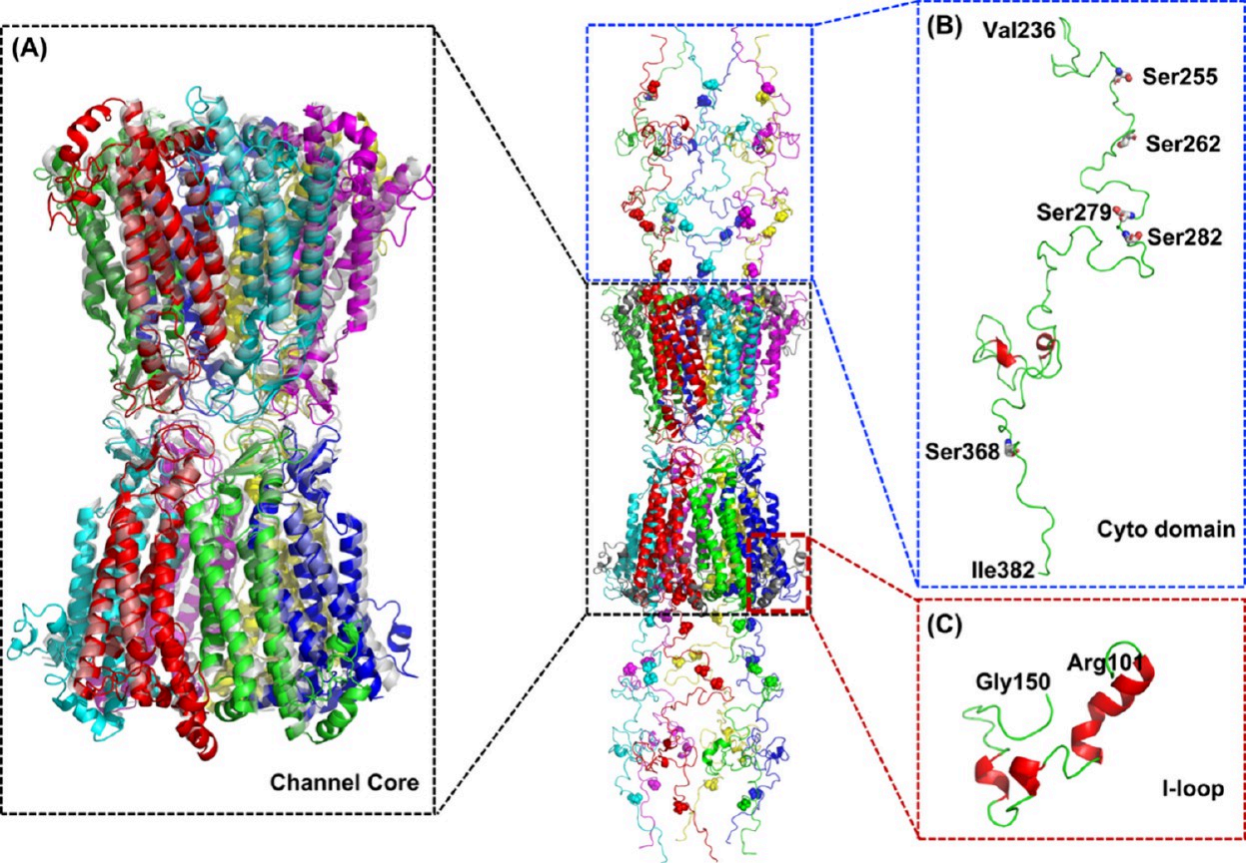
Complete computational Cx43 gap junction channel structure including the intracellular loops and the C-terminal domains. Spheres represent five Ser residues (Ser 255, 262, 279, 282, and 368) that are phosphorylated in vivo. (A) The black boxed structure on the left shows the alignment of the channel core between our constructed model (the transmembrane α-helices of the connexins are shown in different colors) and the shaded gray cryo-EM structure published by Qi et al.^39^ (B) The blue box on the top right shows the C-terminal domain ranging from residue Val236 to the last Cx43 C-terminal residue (Ile382), in which five Ser residues (Ser 255, 262, 279, 282, and 368) of the phosphorylation sites are highlighted. (C) The red box at the bottom right shows the ICL model ranging from residue Arg101 to Gly150. A partially folded α-helix structure is indicated in red.

### Structural and Dynamic Properties of the Junctional Membrane Bilayers

To test the structural stability of the membrane bilayers during simulations in our systems, we monitored the variation of *X* (= *Y*) dimension length (e.g., *XY* = the membrane area) as a function of simulation time (Figure S1A). Each of three replicas in all four systems reached a plateau after ∼1000 ns. The averaged *X* or *Y* dimension length in three replicas of each system is 103, 103, 102, and 102 Å. During the last 500 ns of the simulations, the deviations among each replica were less than 1 Å, indicating no significant differences in the junctional membrane area for both phosphorylated and unphosphorylated systems. Figure S1B shows the distribution profiles of lipid and water molecules along with the membrane normal (i.e., *Z*-axis), in which POPC, POPS, CHOL, and PSM lipids are observed to be located on both sides of *Z* = 0, i.e., the GJC center, and extend to ± ∼70 Å. POPS molecules are observed only in the inner leaflet (i.e., the intracellular side), consistent with the compositions of our membrane leaflets described in Materials and Methods. Consistent with Flores et al. work on Cx46/50 channels,^44^ water molecules were dominantly occupied in the channel center at *Z* = 0 and in the cytoplasmic CTD regions beyond ∼60 Å. Rarely, they were found to be distributed in the membrane bilayers. The distributions of lipids among the four systems are similar to each other (data not shown), indicating that phosphorylation of Ser residues in the CTD does not have detectable effects on the distribution of membrane lipids. However, a slight difference for the distribution of water molecules was detected among the four systems at *Z* = ± ∼90 Å (shown in Figure S2), due to different phosphorylation states of Ser residues. A representative computational channel structure is shown in Figure S1B, in which the channel core helices are surrounded by the lipids, forming a stable GJC conformation.

To investigate the structural stability of the membrane bilayers, the hydrophobic thickness of the two membranes in the two hemichannels (Mem. HC1 and Mem. HC2) as defined by the distance of the C2 and C3 atoms in each lipid between the inner and outer leaflets was calculated. Figure S3A shows the averaged thickness during the last 500 ns of simulations for each system, indicating similar hydrophobic thickness observed among all four systems. The averaged thicknesses of Mem. HC1/Mem. HC2 are 27.00 ± 0.35/26.77 ± 0.23 Å (Cx43-0P), 26.99 ± 0.07/26.23 ± 0.23 Å (Cx43-1P), 27.14 ± 0.14/26.49 ± 0.60 Å (Cx43–3P), and 27.31 ± 0.10/26.40 ± 0.13 Å (Cx43-5P). The similar thickness indicates that phosphorylation of Ser residues in the CTD, as expected, did not cause apparent structural membrane differences, consistent with the distribution probability of lipids along the *Z*-axis as shown in Figure S1B. Thus, intact and stable hexagonal membranes, as shown in Figure S3B, are well maintained during simulations in our systems.

In addition, the lateral diffusion coefficients of phospholipids in two hemichannel membranes were estimated from the slope of the mean-square displacement using the last 200 ns of simulation trajectories (2000 snapshots) for each replica. The calculated diffusion coefficients are summarized in Table S1. The averaged values among the four systems are comparable (1.36 ± 0.52, 1.16 ± 0.90, 1.57 ± 0.86, and 1.33 ± 0.87 μm^2^/s for the Cx43-0P, Cx43-1P, Cx43-3P, and Cx43-5P systems, respectively), indicating similar lateral dynamic diffusion behaviors of bilayers in all systems. It is also indicated that the calculated diffusion constant is smaller than that in one lipid or mixed lipids membrane bilayer, which is usually in 4.5–17.8 μm^2^/s.^71,88^ It is consistent with Lindblom and Orädd,^89^ in which it is indicated that inclusion of 15–20% CHOL to mimic a plasma membrane would increase the packing of the hydrocarbon chains, resulting in a reduced diffusion motion.

### Stable Channel Core and a Flexible C-Terminal Domain

The root mean-square deviation (RMSD) is a measure of the average distance between the atoms of superimposed molecules before and after simulations. Figure 3A shows the computational Cx43-5P structure with the five phosphorylated Ser residues highlighted at the beginning (initial) and at the end of 1500 ns molecular simulations. It is indicated that the channel core conformation is well maintained with stable α-helix bundles consistent with published crystal and cryo-EM structures of GJCs, while the CTDs quickly compress and become more compact during simulations compared to the initial, more straight, and extended conformation that is based on NMR analyses (see Video S1). Figure 3B shows the RMSD variations of backbone atoms compared to the initial structural representation for each GJC system along the simulation time, indicating that the channel core is very stable with low RMSD values of 6.4 ± 0.1 (Cx43-0P), 5.9 ± 0.1 (Cx43-1P), 5.9 ± 0.1 (Cx43-3P), and 5.8 ± 0.1 Å (Cx43-5P). In contrast, consistent with X-ray crystallographic and cryo-EM analyses (i.e., invisible CTD), the CTD is very flexible (due to its loop and random coil conformation) and experiences significant structural changes during simulations, indicated by high RMSD values of 44.1 ± 0.5 (Cx43-0P), 42.1 ± 0.3 (Cx43-1P), 38.4 ± 0.3 (Cx43-3P), and 39.5 ± 0.3 Å (Cx43-5P). Moreover, the slight reduction of RMSD values in phosphorylated Cx43 GJCs may be a result of increased electrostatic repulsion, with more Serine residues phosphorylated in the C-terminal regions, resulting in a more extended C-terminal conformation.

**Figure 3:**
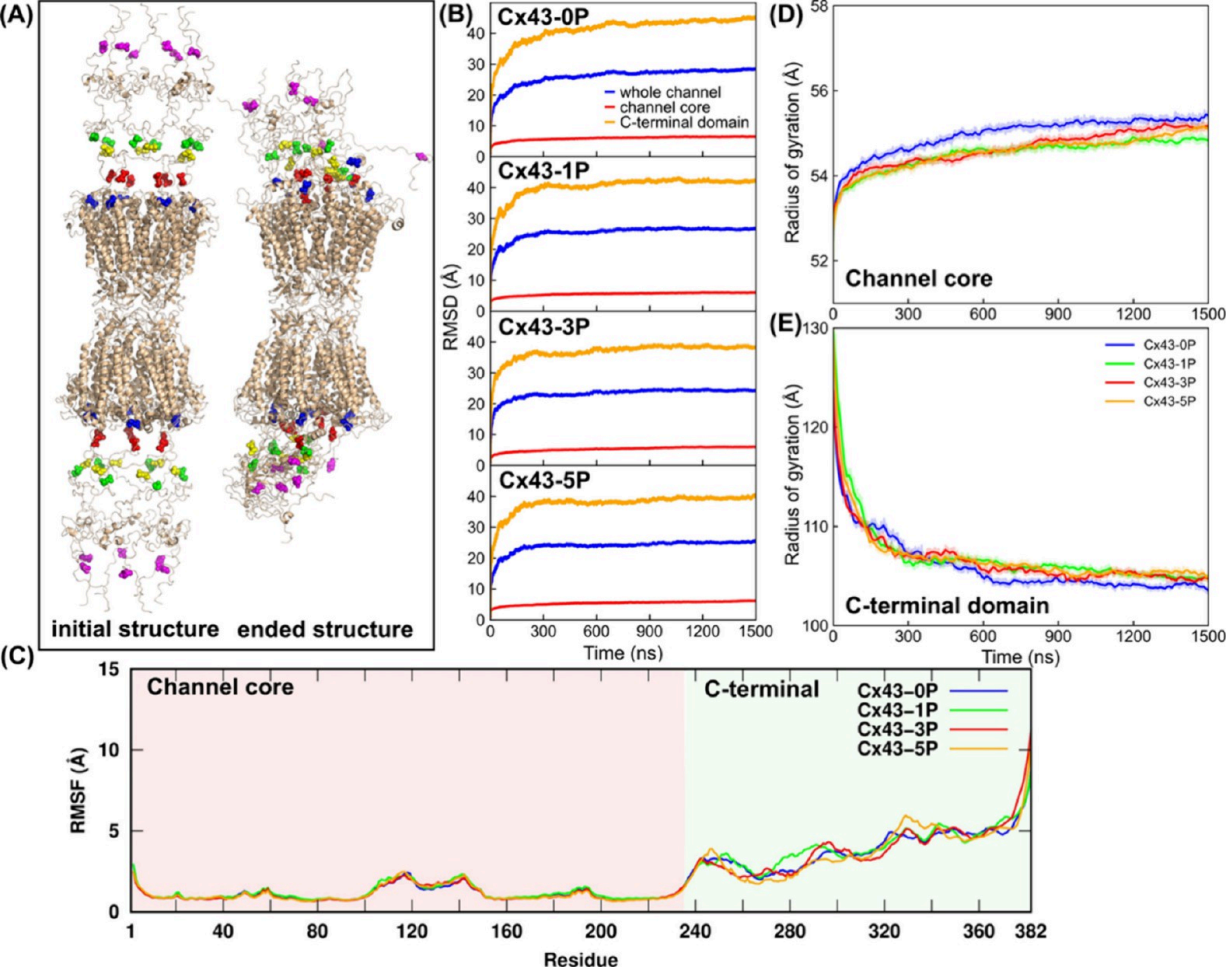
Rigid and compact channel core conformations and flexible, less compact C-terminal domain conformations in phosphorylated Cx43 channels. (A) Computational Cx43-5P GJC structures at the beginning and at the end (after 1500 ns) of molecular simulations. The blue, red, yellow, green, and magenta spheres represent the five phosphorylatable Ser residues, 255, 262, 279, 282, and 368, respectively. Note the significant compaction of CTDs during molecular simulations. (B) Root mean-square deviation (RMSD) variations of the backbone atoms for the whole channel (blue), channel core (red), and C-terminal domain (orange) for unphosphorylated and phosphorylated Cx43 GJCs as a function of simulation time. (C) Root mean-square fluctuations (RMSFs) averaged on 12 connexins in each GJC system. Radius of gyration (Rg) values of (D) the channel cores and (E) the C-terminal domains.

### Less Dense Packed C-Terminal Domain in Phosphorylated Cx43 Channels

The root mean-square fluctuation (RMSF) is a measure of the mean deviation from the average atomic positions over time in molecular dynamic simulations and provides information about the flexibility and dynamics of protein structures. Figure 3C shows the RMSF of the Cx43 protein averaged on two hemichannels (12 connexins) in both phosphorylated and unphosphorylated connexins. The stable channel core conformation (pink shaded area) is indicated by low RMSF values (less than 3 Å) among all four systems. The ICL, ranging from residue 101 to 150, exhibited a slightly higher RMSF fluctuation due to its partially folded α-helix conformation. In addition, well-overlapped RMSF curves in the channel core region indicate a similar dynamic conformation ensemble sampled for all four systems. Interestingly, for the CTD (light-green shaded area), significantly increased fluctuation and flexibility are observed when Ser residues are phosphorylated, which most likely is due to enhanced electrostatic repulsion interactions in the phosphorylated channels. The RMSF analyses agree well with the RMSD results described above, in which also a stable channel core and a flexible CTD conformation were observed.

To further investigate CTD packing of phosphorylated and unphosphorylated Cx43 GJCs, we determined the radius of gyration (Rg), which is an indicator of protein structure compactness. The channel core conformation did not undergo significant changes in packing density, and its Rg fluctuates around 55 Å, as shown in Figure 3D. Furthermore, the channel core conformation in the phosphorylated systems appears slightly more compact compared to the unphosphorylated channels, as indicated by somewhat lower Rg values. In contrast, the CTD Rg shown in Figure 3E decreased rapidly from 130 to 108 Å in the first 200 ns of molecular simulations and gradually reached a value of 104–106 Å in all four systems. Interestingly, slight deviations of Rg values between phosphorylated and unphosphorylated channels were also detected, with higher Rg values of the phosphorylated systems, indicating an overall less compact conformation of the C-terminal domain in phosphorylated Cx43 channels.

### Increased Solvent Exposure to the Intracellular Channel Portion in Phosphorylated Cx43 GJCs

As indicated by our RMSD, RMSF, and Rg analyses, phosphorylation of Ser residues in the CTD results in a more flexible, less compact C-terminal domain conformation, while a rigid and compact channel core conformation is maintained. To elucidate the underlying mechanism of the increased flexibility and less dense C-terminal packing in the phosphorylated channels, the solvent accessible surface area (SASA) of each phosphorylated Ser residue in all four systems was calculated. As shown in Figure 4A, Ser368 appears to be most easily accessible, which correlates with its location closest to the cytoplasmic end of the CTD (see Figure 3), indicated by a higher SASA value compared to any other tested serine residues. In addition, it is shown that the phosphorylation of more than one Ser residue results in an increased exposure to the solvent (water), as indicated by higher SASA values in phosphorylated systems (compare the increase of orange, red, and green bar heights with blue bars when corresponding serine residues are phosphorylated). To be more precise, the exact number of water molecules within 3 Å of residues Ser255, 262, 279, 282, and 368 in each system was counted. Figure 4B shows that the number of water contacts of the individual serine residues increases upon phosphorylation, consistent with the SASA analysis described above (note the increase in bar height with phosphorylation). Taken together, these results confirm that increased phosphorylation leads to an increased SASA with more water molecules around each phosphorylated serine residue, resulting in a more flexible and more dynamic CTD.

**Figure 4:**
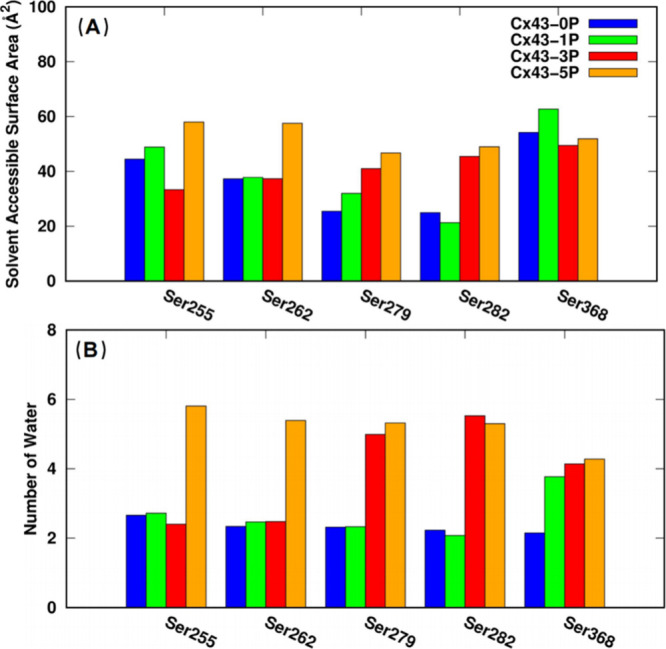
Phosphorylation of Ser residues results in an increased exposure to the intracellular environment. (A) The calculated solvent accessible surface area (SASA) and (B) the number of water molecules within 3 Å for residues Ser255, 262, 279, 282, and 368 in each system. Cx43-0P for no Ser phosphorylation, Cx43-1P for Ser368 phosphorylation, Cx43-3P for Ser279/282/368 phosphorylation, and Cx43-5P for Ser255/262/279/282/368 phosphorylation.

### Electrostatic Potential and Ion Distributions of Cx43 GJCs

Phosphorylation of Ser residues not only increases accessibility to the solvent but also changes the ion distribution and electrostatic potential of the channel. Figure 5A shows the distribution of K^+^ (blue) and Cl^–^ ions (red) in unphosphorylated Cx43 channels (Cx43-0P). The distribution of these ions correlates with the electrostatic potential on the surface of the Cx43-0P GJCs. To visualize and analyze the charge distribution along the entire GJC, in Figure 5B, the channel surface is colored according to its electrostatic potential calculated by ChimeraX.^90^ In both hemichannels, a positive electrostatic potential (shown in blue) in the area where the N-terminal helices (NTH, residues 2 to 19) are positioned (the lipid/cytoplasm boundary) and a neutral to slightly negative potential (shown in white and some red) around the docking region were observed. Thus, as shown in Figure 5C and consistent with Qi et al.,^39^ Cl^–^ ions preferentially accumulate on the channel surface in the lipid/cytoplasm boundary regions as indicated by the anion density peaks around *Z* = ± 60 Å due to electrostatic attraction. At the docking region around *Z* = 0, the density distributions of K^+^ and Cl^–^ ions are comparable, consistent with an observed negative/neutral electrostatic potential of that region. Interestingly, consistent with our previous results on solvent exposure, at the region corresponding to the cytoplasmic portion that is formed by the CTDs (beyond ± 90 Å in Figure 5C) where residues Ser255, 262, 279, 282, and 368 are located, the probability of K^+^ ions increases from 7.8 × 10^–5^ in Cx43-0P to 8.1 × 10^–5^ in Cx43-5P along with more Ser residues phosphorylated. Finally, inside the channel core (about *Z* = ± 15 – ± 50 Å in Figure 5C), consistent with the ion distribution inside the pore observed by Qi et al.,^39^ K^+^ ions dominate.

**Figure 5:**
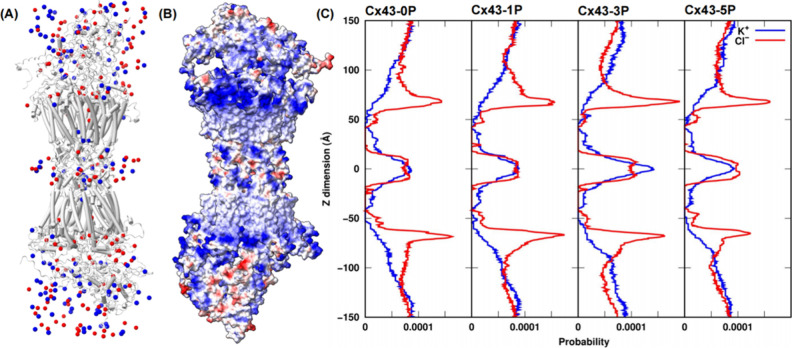
Ions distributions reveal solvent accessible and inaccessible channel regions and increased charge states of the C-terminal regions in phosphorylated Cx43 channels. (A) Ions distribution (blue for K^+^ and red for Cl^–^ ions) in the unphosphorylated (Cx43-0P) GJC. (B) Electrostatic potential along the surface of the Cx43-0P GJC. Red represents negative potential, blue positive, and white neutral potential. (C) Averaged ion density profiles along the *Z*-axis in unphosphorylated and all phosphorylated Cx43 GJCs.

### Channel Pore Properties of Phosphorylated and Unphosphorylated Cx43 GJCs

The gating and permeability of Cx43 GJCs are modulated by various factors, and phosphorylation of Cx43 plays a pivotal role in controlling the opening and closing of GJCs.^15^ The Cx43 channel structure resolved in Qi et al.’s^39^ cryo-EM work was considered to represent a closed channel as the observed positions of the NTH (residues 2 to 19, red in Figure 6A) and TM2 (residues 75 to 105, blue in Figure 6A) resulted in a channel with a minimum solvent-accessible radius (the gate) of only ∼3 Å (average solvent accessible radius of the entire channel was ∼6 Å), making the Cx43 GJC the narrowest pore of all GJ channels investigated to date. Lee et al.^38^ performed cryo-EM single particle analyses of Cx43 GJCs under various conditions and identified three distinct NTH gate conformations termed gate-covering (GCN), pore-lining (PLN), and flexible intermediate (FIN) that coexisted in the purified channel populations. However, in both structures from Lee et al.^38^ and Qi et al.,^39^ the C-terminal domains were not resolved.

**Figure 6:**
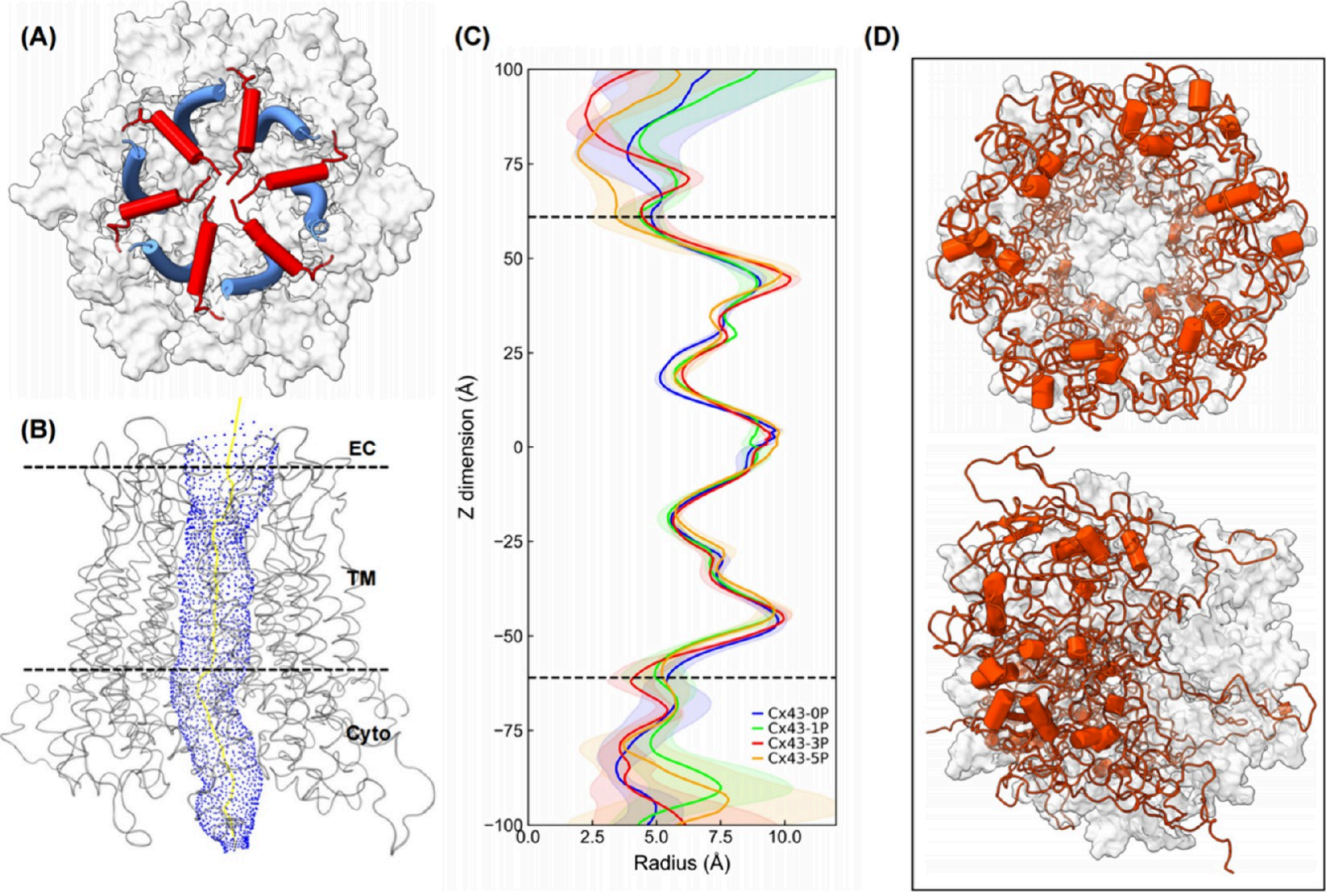
Phosphorylation of connexins narrows the pore of the channel. (A) Top view of Cx43 channel gating in our computational structure seen from the cytosolic side. The Cx43-0P channel at beginning of MD simulations is shown as a representative example. For clarity, the C-terminal domains were removed. The gate-forming regions of the pore, N-terminal helices (NTHs; red) and TM2 (blue), are shown as cylinders. The white surface corresponds to the rest of the protein. (B) A visualization of the channel pore (blue) formed by Cx43 including the C-terminal domains using the program HOLE. (C) Channel radii along the *Z*-axis of all systems. The dotted lines correspond to the positions of the NTHs. (D) Structural views of connexin gating states for Cx43-0P GJC before (top) and after (bottom) MD simulation seen from the cytosolic side. The C-terminal regions are represented as red, and the remaining regions are represented as the white surface. Note the occlusion of the pore entrance.

To investigate how the hydrophilic channel (the channel pore) appears in a complete Cx43 GJC, we analyzed the pore characteristics of unphosphorylated and phosphorylated Cx43 GJCs. A visualization of the Cx43-5P hemichannel pore including its complete cytoplasmic portion generated using the program HOLE^91^ is shown in Figure 6B (shaded in blue) as a representative. The averaged radius of the entire channel pore for all four systems is shown in Figure 6C (the last 500 ns trajectories of three replicas were used for these analyses). The shaded region represents the standard error of the mean. It shows that the closed gating state in the cryo-EM structure of Qi et al. experiences a drastic conformational change during MD simulations, indicated by the non-negligible fluctuations at the NTD position around ±60 Å. The averaged pore radius at the vestibule (the narrowest point of the pore) of two hemichannels (HC1/HC2), defined by the region formed by NTHs and TM2, is 5.4 ± 0.7 Å/4.8 ± 0.3 (Cx43-0P), 4.9 ± 1.3 Å/4.3 ± 0.9 (Cx43-1P), 4.0 ± 0.9/4.4 ± 0.9 Å (Cx43-3P), and 5.2 ± 0.7/3.4 ± 0.9 Å (Cx43-5P), which is slightly smaller than that in Qi et al.’s^39^ work. Of note, the two connexons of a channel do not exhibit the exact same dynamic conformation due to their dynamic fluctuations and are indicated by different pore radii at the vestibule. Interestingly and unexpectedly, a somewhat narrower pore radius was detected when more Ser residues were phosphorylated. The region beyond *Z* = ± 60 Å represents the channel portion formed by the CTDs. As expected, it exhibits a diverse and fluctuating gating state, which is consistent with the flexible structural properties of the CTD that we characterized before. A comparison from the cytoplasmic view between the initial and final complete Cx43-5P conformation is shown in Figure 6D and Video S2. It shows that the channel entrance is occluded by the C-terminal regions after simulations. However, this may be due to unrestrained movements of the CTDs in our computationally limited single-channel simulations that are not spatially restricted by the CTDs of neighboring plaque GJCs. Lampe and Lau^21^ reported that PKC-mediated phosphorylation at Ser368 induces a shift in unitary conductance from a full open state to a lower-conductance state, leading to decreased gap junction communication. In addition, in Warn-Cramer et al.,^92^ it is indicated that phosphorylation on Ser255, Ser279, and Ser282 initiates downregulation of gap junctional communication, and triple mutations of MAPK sites rendered the channel resistant to growth factor-induced disruption. Our simulated transition to a narrower gating state upon phosphorylation aligns with these experimental findings, reinforcing the role of the CTD as a vital regulatory element that exerts long-range allosteric control over channel gating and permeability.

### Modulation of Cx43 Channel Gating by CTD Phosphorylation

To understand how structural changes of the CTDs incurred by phosphorylation that we observed could impact the conformation of the channel core and, e.g., its molecular gate that is located far away from the cytoplasm, we calculated the correlation of residue fluctuations to explore the importance of collective motions for Cx43 GJCs. The different gating states of Cx43 GJCs in different phosphorylation conditions are proposed to be closely associated with the dynamic interaction between NTHs and TM2,^38^ which is indicated by intramolecular hydrophobic interactions formed by Leu10 and Val14 in NTH, and Tyr92, Leu93, Phe97, and Met100 in TM2 ^38^ (shown in Figure 7A and Video S3). The NTHs are short helices arranged horizontally at the entrance of the pore in the cryo-EM structure. However, these short helices experienced drastic structural fluctuations during our MD simulations in the different systems. The averaged helical population of each connexin for each system in each replica during the last 500 ns simulations is summarized in Table S2. While most NTHs maintained a well-structured helical conformation, some connexins, as for example the sixth connexin in HC1 of Cx43-0P and the third connexin in HC2 of Cx43-1P in replica 1, adopted only very little helical conformation, indicating instead an unfolded N-terminal domain. The averaged helical population of NTHs in two HCs of Cx43-0P, Cx43-1P, Cx43-3P, and Cx43-5P is summarized in Figure 7B. Slight, yet significant differences among the four systems were detected. In average, the helical population of NTHs in Cx43-3P and Cx43-5P systems was higher than those in Cx43-0P and Cx43-1P systems, especially for the NTHs in Cx43-3P HC1 (blue bar) and Cx43-5P HC2 (red bar) (helical populations are 60.7% and 63.6%, respectively), corresponding to a narrower pore radius (4.0 ± 0.9 and 3.4 ± 0.9 Å) in these systems. Figure 7C shows the secondary structure analysis for Cx43-0P HC1, in which, as described above, the NTH of the sixth connexin is almost unfolded and, instead, during the last 500 ns simulations adopts a β-bend or β-turn structure. The unfolding of the NTH led to a disruption of its hydrophobic interactions with TM2, resulting in a distorted gating pore (shown in Figure 7D, top). The initial closed state is transitioned to a more open gating state, compared to Cx43-5P HC2, in which all NTHs are well retained (Figure 7D, bottom).

**Figure 7:**
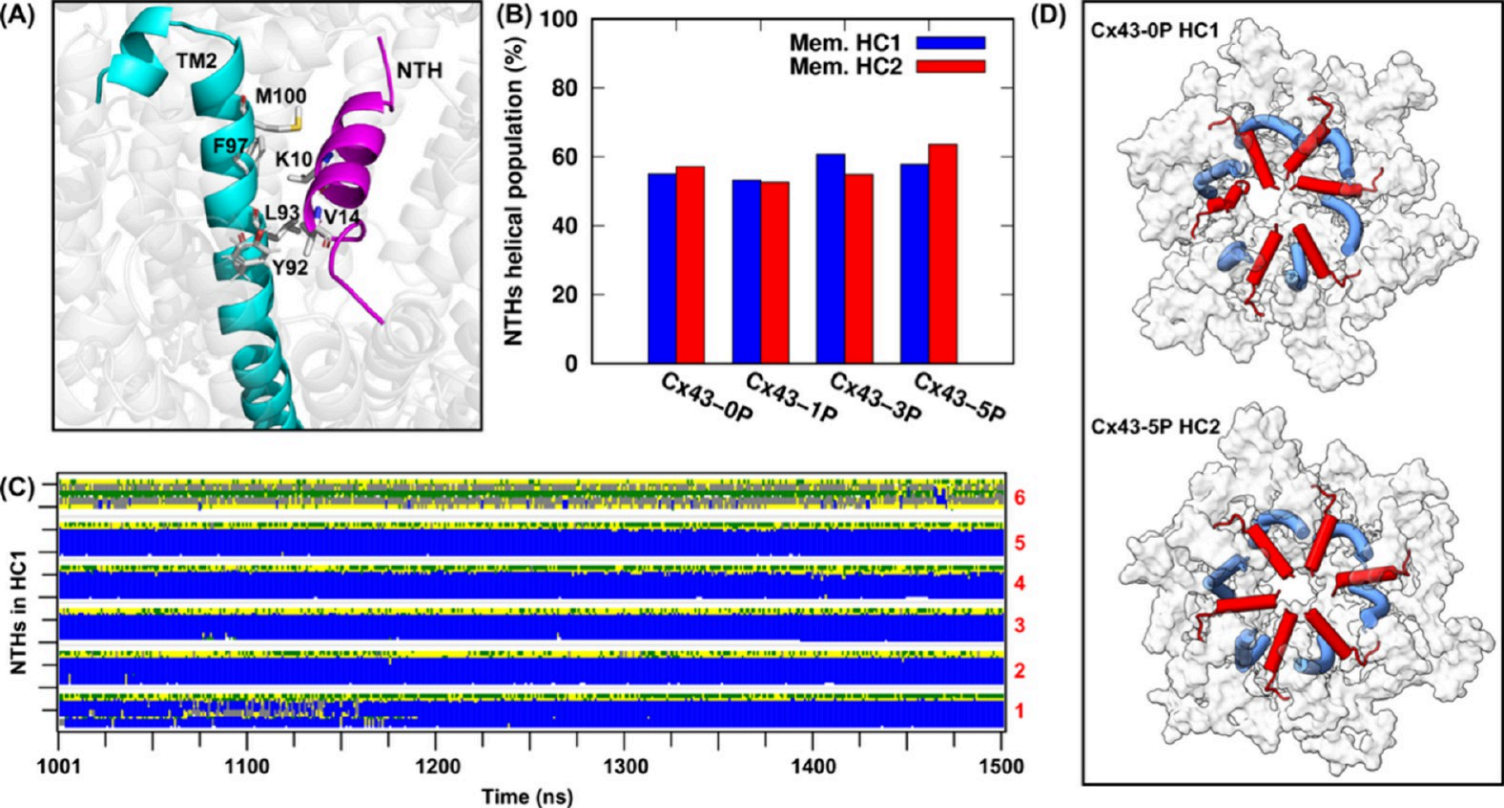
Disrupted hydrophobic interactions between NTHs and TM2 results in a more open gating state. (A) Hydrophobic interactions formed between NTH and TM2 in Cx43 GJCs. (B) Averaged helical population of NTHs in each hemichannel (HC) for each system. (C) Secondary structure analysis for NTHs 1 to 6 in the Cx43-0P HC1 system during the last 500 ns of simulations shown as an example. α-Helices are colored blue, β-bends green, β-turns yellow, 3_10_-helices gray, and coils white. (D) A structural view of connexin gating states for the Cx43-0P HC1 and Cx43-5P HC2 systems from the cytosolic side. The gate-forming regions, NTHs (red) and TM2 (blue), are shown as cylinders. The white surface corresponds to the rest of the protein. The C-terminal regions are excluded for clearer representation.

### Role of Collective Molecular Motions in CTD Phosphorylation and Gating Modulation

To further investigate the internal dynamics of Cx43 GJCs, especially the dynamic relationship between the pore gating and the CTD, and to explore the importance of collective molecular motions, we calculated the correlation of residue fluctuations. The dynamic cross-correlation between residues *i* and *j*, *C*_*ij*_, is given by $C_{ij}=<\Delta r_{i}\cdot \Delta r_{j}>/\sqrt{<\Delta r_{i}^{2}><\Delta r_{j}^{2}>}$ , where Δ*r*_*i*_ is the instantaneous displacement of residue *i* from its average position. The cross-correlation values range from +1 (perfectly correlated motion) to −1 (perfectly anticorrelated motion). Positive values indicate that the residues move in the same direction; negative values indicate movement in opposite directions. We found that the averaged cross-correlation map of each HC in each GJC system is similar. In Figure 8A, the cross-correlation map of one HC in the Cx43-5P GJC system is shown as an example. Clearly, residues in the TM helices are positively correlated, exhibiting a coordinated overall motion and thus maintaining the integrity of the channel core conformation. In contrast, for the CTD region (residues 237 to 382), anticorrelated movements with the channel core region (residues 2 to 236), indicated by the blue-colored bottom right corner, are observed. Therefore, increased phosphorylation of serine residues in the CTD enhances electrostatic repulsion, leading to a straighter, more extended, and flexible CTD conformation. This, in turn, promotes a more compact arrangement of the channel core, which is consistent with the results of our Rg analyses (Figure 3C).

**Figure 8:**
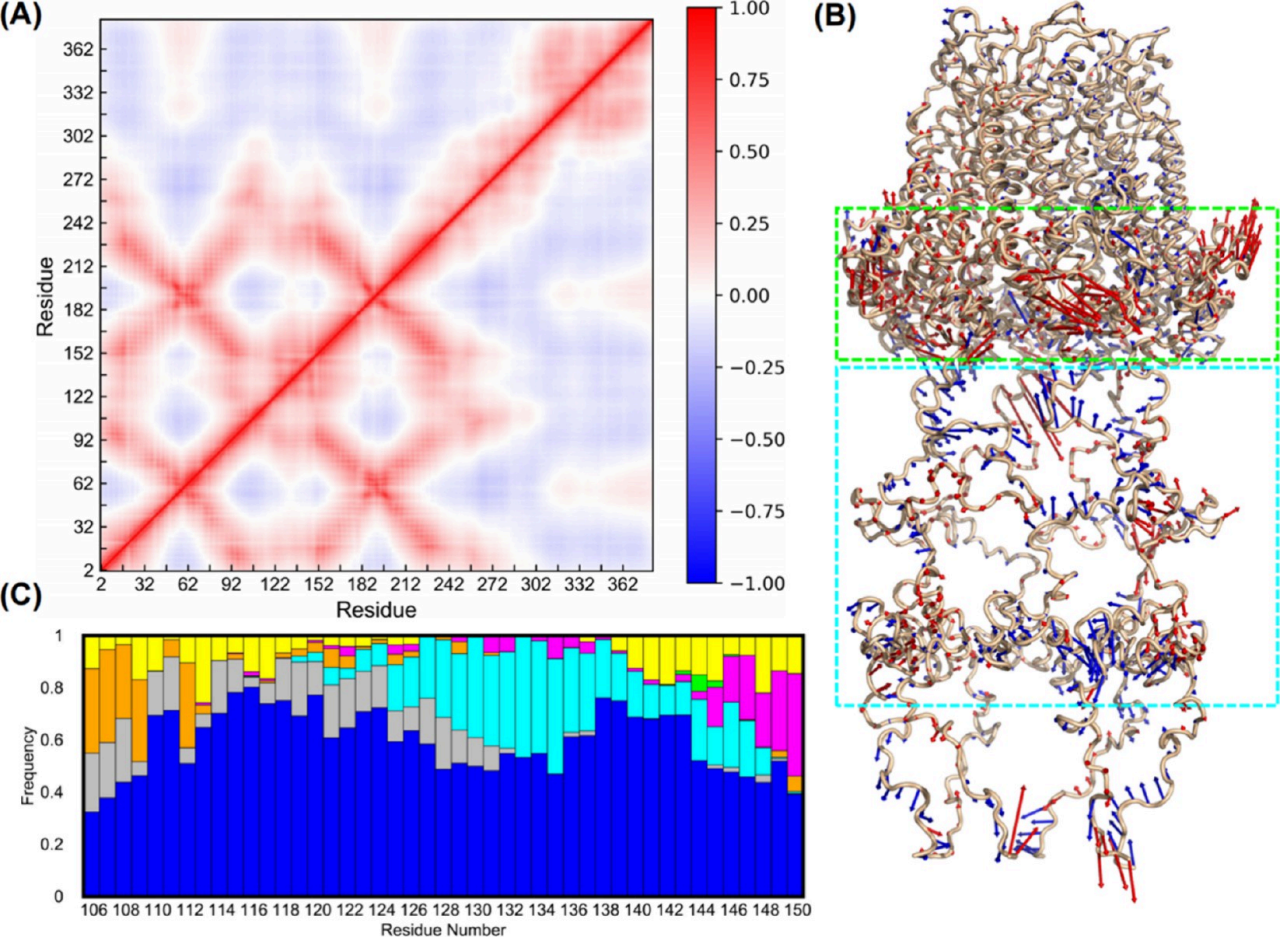
Anticorrelated motions between the C-terminal domain and gating region indicate dynamic coupling in Cx43-5P GJCs. (A) Representative averaged dynamic cross-correlation map calculated using the Cα atoms for one HC in the Cx43-5P system. (B) Concerted motions of the Cx43-5P HC are represented by the first principal component (PC1). Arrows indicate the direction and relative magnitude of correlated displacements of Cα atoms, with red arrows showing motions along the positive direction of PC1 and blue arrows showing motions along the negative direction. The CTD and the near-NTH regions exhibiting motions in opposite directions along PC1 are shown in cyan and green boxes, respectively. The cartoon trace of the protein backbone is overlaid to highlight the structural context of these dominant collective motions. (C) Contact frequencies of the intracellular loop (ICL) residues (106–150) with their surrounding environments averaged over 12 connexin monomers in Cx43-5P GJCs: water (blue), another Cx monomer (gray), lipids (cyan), TM2 (orange), TM3 (magenta), TM4 (green), and CTD (yellow). A contact is counted when the distance between any heavy atom of a residue and that of its interacting partner is less than 4 Å. Contacts are normalized for each interacting partner.

In addition, to capture the major structural variations and dynamic behaviors of the GJC, a principal component analysis (PCA) was conducted for the Cx43-5P HC using the last 200 ns trajectories. The concerted motions of the Cx43-5P hemichannel as captured by the first principal component (PC1) are illustrated as an example in Figure 8B. The short red arrows on the TM helices indicate that these regions exhibit minimal motion along PC1, suggesting that the TM domains are relatively rigid and experience limited conformational changes during simulations. However, the CTD region (cyan box) and the near-NTH region (green box) exhibit motions in opposite directions along PC1, indicated by predominantly blue arrows in CTD and red arrows in the near-NTH region. The blue arrows in the CTD suggest movements along the negative direction of PC1, while the red arrows near the gate represent displacements along the positive direction. This opposing motion pattern implies an anticorrelated dynamic relationship, and fluctuations of the CTD may be mechanically or dynamically coupled to conformational changes near the gating region, potentially contributing to the regulation of channel opening and closing, which is consistent with the dynamic cross-correlation analysis. Collectively, the analyses described above may explain how phosphorylations in the CTDs affect the gating characteristics of GJCs. Experimentally, FRET-based assays could be employed to measure real-time distance changes between these domains upon phosphorylation. In addition, dual-cell patch-clamp electrophysiology using relevant phosphorylation mimics and mutants could confirm whether the narrowing of the pore radius observed in our study indeed results in the expected reduction of junctional conductance.

### Structural Stability and Environmental Contacts of the ICL Domain

To also explore the interaction patterns of the ICL domain (red box in Figure 2), which is also not resolved in the cryo-EM structures but was modeled here using CHARMM-GUI with its surrounding environments, contact frequency of each ICL residue averaged over 12 monomers in two HCs with lipid molecules, water, adjacent monomers, TMs 1–4, and CTD in the monomer was calculated (Figure 8C). A distance cutoff of 4 Å was used to define a contact between any heavy atom of each ICL residue and that of each environmental moiety. It is indicated that multiple and diverse interactions between ICL residues and almost all tested environments are formed during simulations that affect the structural and functional properties of the ICL domain and the channel as a whole. Interestingly, while all ICL residues can form contacts with water, certain ICL domain sections favor distinct interactions. For example, residues Leu106 to Thr118, which are located juxtaposed to TM2 form significant interactions with that helix, the CTD, and an adjacent Cx monomer. In contrast, residues Asp119 to Glu140 that resemble the central region of the ICL domain interact preferentially with lipids and thus appear to be in close contact with the membrane bilayer. This finding may explain why almost all known interactions of Cx43 with other binding partners characterized today occur via its CTD, despite the presence of 11 lysines as well as a significant number of charged residues in the ICL domain.^59^ Finally, residues Glu141-Gly150 located juxtaposed to TM3 interact significantly with that helix, the CTD, and to some extent with lipids. Only very limited interactions were detected with TM4. TM1 is separated from the ICL domain by TM2 and TM3, and no interactions between TM1 and the ICL domain were observed. The interaction patterns of the ICL domain are relatively stable throughout the entire simulation time, suggesting that the ICL domain does not experience significant structural fluctuations over time and hence can be considered as structurally stable.

## Conclusions

Connexin-43 gap junction channels (Cx43 GJC) play a crucial role in diverse biological processes by providing a direct pathway for electrical and metabolic signaling between cells. Mutations or dysregulation of Cx43 is associated with various human diseases. The gating and permeability of the Cx43 GJC are modulated by multiple factors, among which phosphorylation plays a pivotal role. However, the molecular basis underlying the function and regulation of Cx43 GJCs remains incompletely understood, largely due to the lack of a high-resolution structure of the complete channel. Significant progress on solving the structure of vertebrate (connexin-based) and invertebrate (innexin-based) GJ channels and of related pannexin channels at atomic resolution has been made over the past years, mainly based on the successful reconstitution of GJ proteins in lipid nano discs,^93^ single-molecule cryo-EM analyses,^38,39,44–50,56,57^ and the availability of radically improved AI-based 3D-protein folding algorithms such as AlphaFold2.^94^ However, due to remaining experimental limitations, none of the structures available today include the intrinsically unstable, yet important regulatory C-terminal domain (CTD), nor the intracellular loop (ICL) domain.

Here, we present for the first time, the molecular representation of a complete Cx43 gap junction channel including the previously unsolved ICL and CTD, based on the cryo-EM-based atomic resolution structure of the channel core solved by Qi et al.,^39^ and the lowest energy 3D solution NMR structure of the C-terminal domain published by Sorgen et al.^60^ To elucidate the effects of C-terminal phosphorylation on the structure and function of Cx43 GJCs, three systems representing different phosphorylation states, Cx43-1P (Ser368 phosphorylated), Cx43-3P (Ser368/Ser279/Ser282 phosphorylated), and Cx43-5P (Ser368/Ser279/Ser282/Ser262/Ser255 phosphorylated), all phosphorylations that have a significant function in the endocytosis and turnover of GJCs, besides the unphosphorylated channel (Cx43-0P), were modeled. All-atom MD simulations were performed with all systems as well to investigate the structural dynamics and functional properties of the channels. The simulations reveal that the system membrane area and the distribution probabilities of lipids and water molecules are similar across all four systems. The hydrophobic thicknesses of the two membranes are also comparable, and stable hexagonal membrane architectures are well maintained throughout the simulations for all systems. Analysis of the structural dynamics shows that the channel core, consistent with previous analyses by others, remains stable, with RMSDs fluctuating around 6.0 Å. In contrast, the CTD exhibits significant flexibility due to its loop and random coil structure and undergoes notable conformational changes. Clear differences in RMSD, RMSF, and Rg among the four different phosphorylation states are observed, suggesting that the phosphorylation of serine residues induces a less dense packing and more extended conformation of the CTD, consistent with increased hydration. This finding is in agreement with reported structural changes of the Cx43-CTD that occur upon phosphorylation/dephosphorylation of serines 365/368 that prevent PKC-mediated downregulation of GJIC,^95^ and our hypothesis that phosphorylation of the CTD (including the five serine residues tested here) and associated loosening of CTD packing is required to permit enzymes that modify GJ channel function throughout their live-cycle, such as kinases, phosphatases, ubiquitinases, and endocytosis machinery components such as AP-2, other CLASPs, and clathrin itself, to sterically access their respective binding sites in the relative densely packed, regulatory CTD.

Furthermore, the phosphorylation of connexins results in narrowing of the channel pore, as indicated by a decreased pore radius. Consistent with previous results, the various gating states of Cx43 GJCs also in the different phosphorylation states are closely associated with hydrophobic interactions between NTH and TM2. Interestingly, the NTHs exhibit distinct structural fluctuations during MD simulations in the different phosphorylation systems. In particular, the unfolding of NTH disrupts hydrophobic interactions with TM2, resulting in distortion of the gating pore and a transition from the initial closed state to a more open gating conformation.

Lastly, we report that the ICL domain maintains numerous contacts with various environments including other channel domains (e.g., TMs and CTD), water, and lipids and can be considered structurally stable. Overall, our work provides novel and comprehensive insights into the structural and dynamic properties of an entire Cx43 GJC, with a particular focus on the effects of CTD phosphorylation. As phosphorylation of Cx43 is well-known to play an important role in Cx-related diseases^15,96–98^ and in particular cardiac function,^95,99–102^ our work offers significant insights into rational drug design, enabling the screening of small molecules or peptides that could prevent detrimental GJ loss, stabilize the open gating state, or prevent the deleterious pore narrowing induced by hyperphosphorylation. In addition, targeting the identified allosteric communication between the distal CTD and the NTH/TM2 gating region provides a novel therapeutic strategy to modulate the GJ channel permeability in clinical contexts. These findings further advance our understanding of the molecular mechanisms governing Cx43 GJC function and regulation in health and disease.

## Supplementary Material









## Data Availability

Data are available in the article itself and its Supporting Information. Initial conformations for our four systems have been uploaded to GitHub. (https://github.com/graceYaGao/Cx43-GJC). Other relevant files are available upon request.

## Notes

Additional data, including lateral diffusion coefficients of phospholipids, averaged helical population of NTHs, *X*/*Y* system dimensions and distribution probability of lipid and water molecules, density profiles for water molecules along the membrane normal, hydrophobic thickness of the membrane, and videos of molecular dynamics simulation of the Cx43-5P gap junction channel (PDF)
Side view of molecular dynamics simulation of the Cx43-5P gap junction channel as a representative example (all lipids were removed; each connexon is depicted as cartoon and colored white; the corresponding NTHs, TM2, and CTDs are colored with red, blue, and cyan, respectively) (MP4)
Top view of molecular dynamics simulation of the Cx43-5P gap junction channel as a representative example (all lipids were removed; each connexon is depicted as cartoon and colored white; the corresponding NTHs, TM2, and CTDs are colored with red, blue, and cyan, respectively) (MP4)
Top view of molecular dynamics simulation of the Cx43-5P gap junction channel as a representative example (all lipids and the C-terminal domains were both removed; each connexon is depicted as cartoon and colored white; the corresponding NTHs and TM2 are colored with red and blue, respectively) (MP4)
