# Supplementary material for: Molecular Modeling and Dynamics of a Complete Connexin-43 Gap Junction Channel in Various Phosphorylation States
Source: J Phys Chem B. 2026 Mar 16;130(12):3384–97. doi: 10.1021/acs.jpcb.6c00338 (PMC13034422; doi:10.1021/acs.jpcb.6c00338)
Supplement: Supplementary file 1 [file jp6c00338_si_001.pdf]

Supporting Information

Molecular Modeling and Dynamics of a Complete Connexin-43 Gap Junction Channel in Various Phosphorylation States

Ya Gao[1,2], Jian Zuo[1], Matthias M. Falk[2*], and Wonpil Im[2*]

[1]School of Mathematics, Physics and Statistics, Shanghai University of Engineering Science, Shanghai 201620, China

[2]Department of Biological Sciences, Lehigh University, Bethlehem, PA 18015, USA

*Corresponding author e-mail: mmf4@lehigh.edu and wonpil@lehigh.edu

Table S1. Lateral diffusion coefficients of phospholipids in each replica for each system.

		Diffusion Coefficient ($\mu m^2/s$)			
		Cx43-0P	Cx43-1P	Cx43-3P	Cx43-5P
Replica 1	Mem. HC1	1.35	1.32	2.77	0.58
	Mem. HC2	1.94	0.10	1.68	0.86
Replica 2	Mem. HC1	1.50	1.39	0.74	2.94
	Mem. HC2	1.52	0.23	1.31	0.91
Replica 3	Mem. HC1	0.38	2.54	2.31	1.68
	Mem. HC2	1.45	1.38	0.60	1.01
**Average**		**1.36 ± 0.52**	**1.16 ± 0.90**	**1.57 ± 0.86**	**1.33 ± 0.87**

Table S2. Averaged helical population of NTHs in two hemichannels for each system.

Replica			Cx43-0P	Cx43-1P	Cx43-3P	Cx43-5P
1	HC1	Cx43-1	61.3	51.9	66.6	56.7
		Cx43-2	66.4	57.3	67.9	65.5
		Cx43-3	62.7	38.9	66.0	66.6
		Cx43-4	64.8	64.9	30.0	9.2
		Cx43-5	66.2	66.4	66.5	65.0
		Cx43-6	1.6	66.8	66.5	51.4
		**Average**	**53.8**	**57.7**	**60.6**	**52.4**
	HC2	Cx43-1	65.6	32.1	65.4	66.1
		Cx43-2	63.5	60.7	54.8	67.3
		Cx43-3	66.0	14.3	30.1	66.8
		Cx43-4	57.9	49.2	66.3	61.8
		Cx43-5	42.9	52.5	51.3	66.8
		Cx43-6	61.3	59.7	36.9	66.0
		**Average**	**59.5**	**44.7**	**50.8**	**65.8**
2	HC1	Cx43-1	55.2	47.3	66.0	66.6
		Cx43-2	65.0	53.4	66.5	66.6
		Cx43-3	66.3	66.1	65.1	64.9
		Cx43-4	55.1	60.8	60.5	58.9
		Cx43-5	45.7	8.7	56.0	38.2
		Cx43-6	49.7	65.0	66.3	62.7
		**Average**	**56.2**	**50.2**	**63.4**	**59.6**
	HC2	Cx43-1	58.6	62.0	4.9	66.3
		Cx43-2	55.1	64.9	65.4	66.0
		Cx43-3	61.0	66.0	66.1	65.0
		Cx43-4	56.9	66.0	66.4	60.4
		Cx43-5	36.4	37.6	30.0	44.9
		Cx43-6	67.0	63.3	65.2	67.0
		**Average**	**55.8**	**60.0**	**49.7**	**61.6**
3	HC1	Cx43-1	57.8	55.9	60.0	65.2
		Cx43-2	50.2	66.1	65.6	65.3
		Cx43-3	39.6	56.1	61.1	66.5
		Cx43-4	55.4	56.0	52.5	66.1
		Cx43-5	54.2	59.3	62.1	40.8
		Cx43-6	72.0	16.9	48.1	64.9
		**Average**	**54.9**	**51.7**	**58.2**	**61.4**
	HC2	Cx43-1	56.9	55.9	65.8	68.2
		Cx43-2	58.6	66.8	64.2	66.4
		Cx43-3	32.9	64.7	66.6	60.9
		Cx43-4	58.2	62.0	66.1	57.2
		Cx43-5	65.8	35.3	57.2	62.6
		Cx43-6	65.6	33.2	65.3	65.0
		**Average**	**56.3**	**53.0**	**64.2**	**63.4**

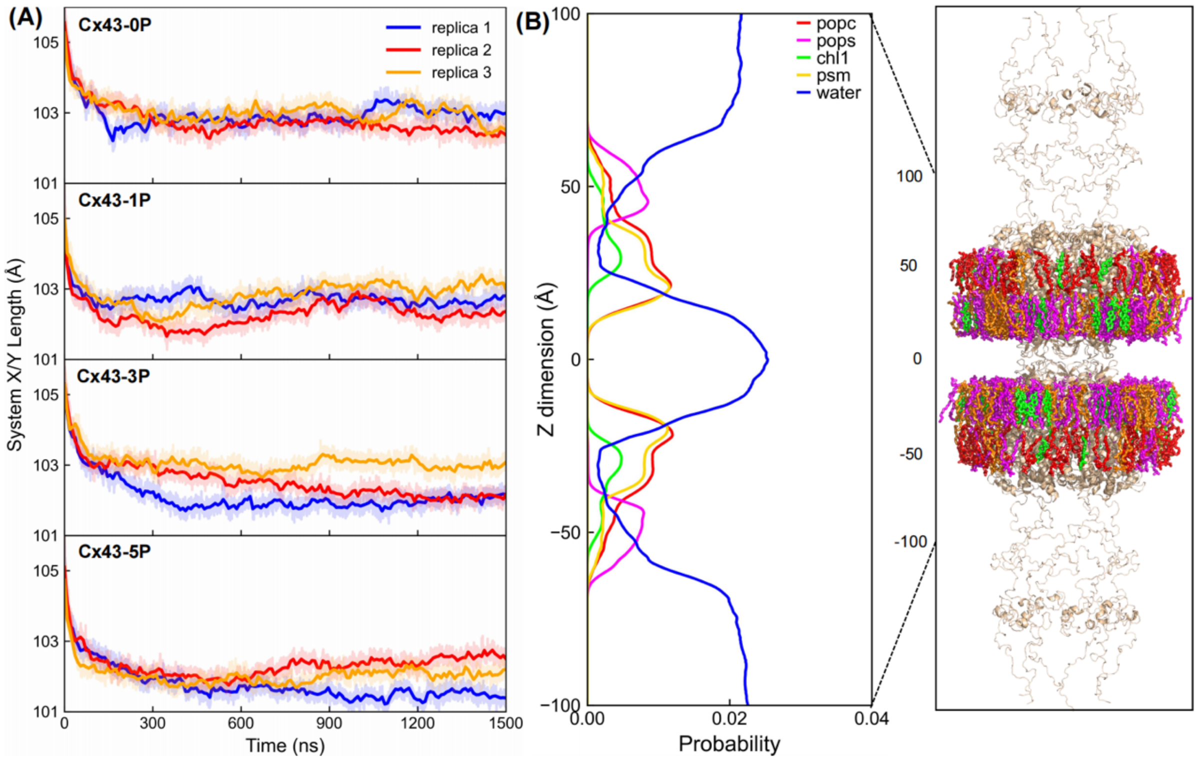

Figure S1. X/Y dimensions and distribution probability of lipid and water molecules in the phosphorylated and unphosphorylated systems were similar in our models. (A) Variations of system X or Y dimension (XY = membrane area) as a function of simulation time across three replicas. **(B)** Density profiles along the membrane normal (i.e., Z-axis) for POPC, POPS, PSM, CHOL, and water molecules. A Cx43-0P gap junction channel structure is shown. Phosphorylation systems are not shown as phosphorylated and unphosphorylated systems exhibit essentially identical lipid and water molecule distribution profiles. The protein was colored in wheat, and POPC, POPS, PSM, CHOL were colored in orange, red, magenta, and green, respectively.

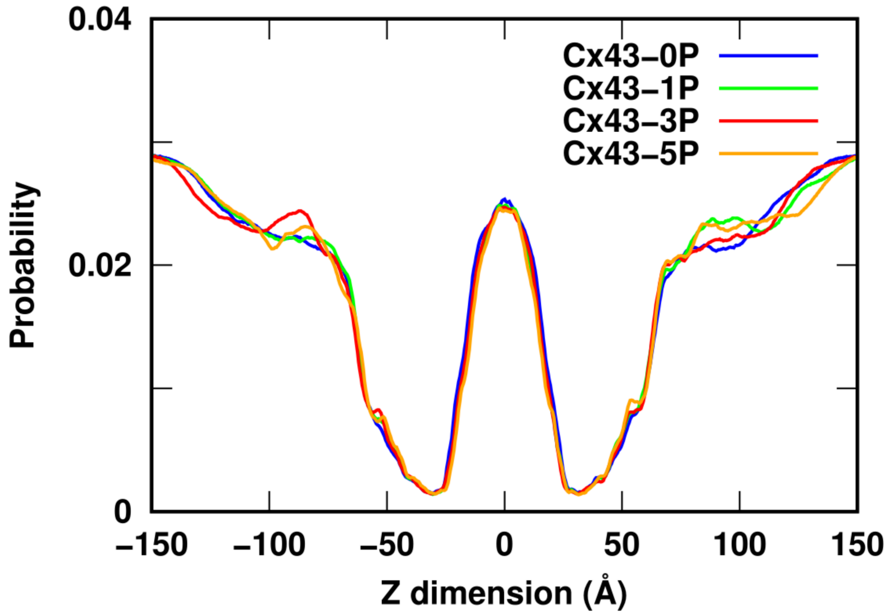

Figure S2. Density profiles along the membrane normal (i.e., Z-axis) for water molecules in each system.

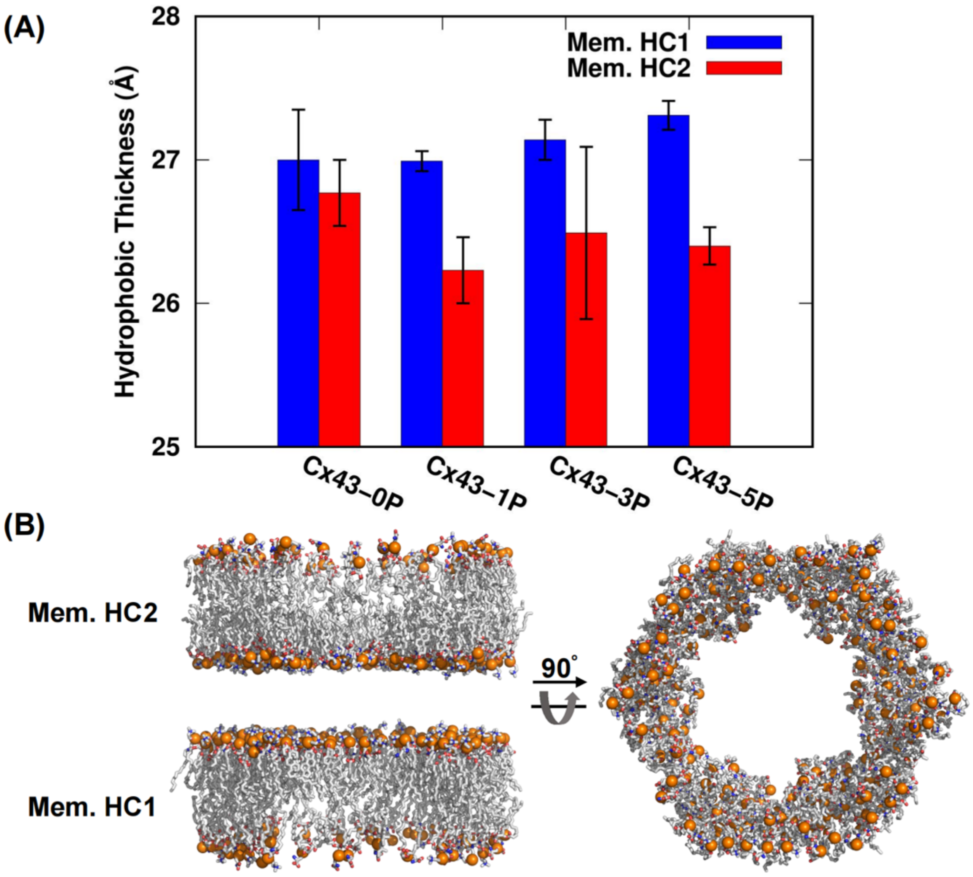

Figure S3. Hemichannel membranes have similar hydrophobic thickness. (A) Averaged hydrophobic thickness of the two membranes of two opposed hemichannels for unphosphorylated and phosphorylated channel structures. **(B)** Membranes of the Cx43-0P GJC are shown as an example.

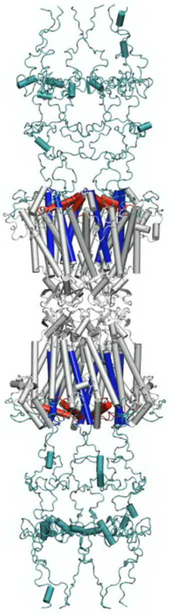

Video S1. Side view of molecular dynamics simulation of the Cx43-5P gap junction channel as a representative example. For clarity, all lipids were removed. Each connexon are depicted as cartoon ad colored white. The corresponding NTHs, TM2, and CTDs are colored with red, blue, and cyan, respectively.

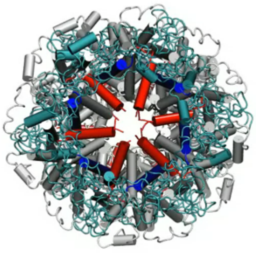

Video S2. Top view of molecular dynamics simulation of the Cx43-5P gap junction channel as a representative example. For clarity, all lipids were removed. Each connexon are depicted as cartoon ad colored white. The corresponding NTHs, TM2, and CTDs are colored with red, blue, and cyan, respectively.

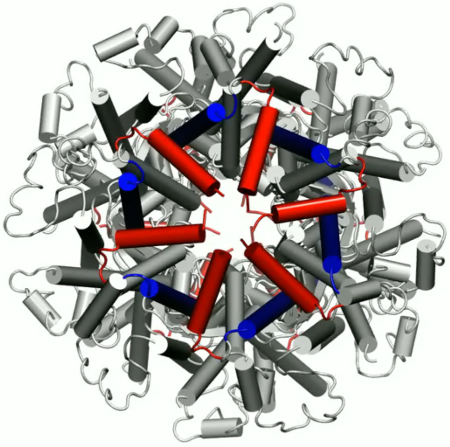

Video S3. Top view of molecular dynamics simulation of the Cx43-5P gap junction channel as a representative example. For clarity, all lipids and the C-terminal domains were both removed. Each connexon are depicted as cartoon ad colored white. The corresponding NTHs and TM2 are colored with red and blue, respectively.
